# Supplementary material for: Three-dimensional X-ray visualization of axonal tracts in mouse brain hemisphere
Source: Sci Rep. 2016 Oct 11;6:35061. doi: 10.1038/srep35061 (PMC5057144; doi:10.1038/srep35061)
Supplement: Supplementary Information [file srep35061-s2.pdf]

Supplementary information for:

Three-dimensional X-ray visualization of axonal tracts in mouse brain hemisphere

Ryuta Mizutani[1,*], Rino Saiga[1], Masato Ohtsuka[2], Hiromi Miura[2], Masato Hoshino[3], Akihisa Takeuchi[3], and Kentaro Uesugi[3]

[1]Department of Applied Biochemistry, Tokai University, Hiratsuka, Kanagawa 259-1292, Japan; [2]Tokai University School of Medicine, Isehara, Kanagawa 259-1193, Japan; [3]Japan Synchrotron Radiation Research Institute (JASRI/SPring-8), Sayo, Hyogo 679-5198, Japan;

*corresponding.author: ryuta@tokai-u.jp

Supplementary Video Legend

Supplementary Video S1. Three-dimensional rendering of the left hemisphere of murine brain. Linear attenuation coefficients from 0.3 cm^{-1} to 3.5 cm^{-1} were rendered with gray scale. First, the entire hemisphere is rotated along the sagittal axis to show its overall structure. Then, coronal cutaways are shown by moving the section plane from the olfactory bulb to the medulla. Subsequently, the section plane goes back and the entire hemisphere returns to the original position.
